# The *Mystery Arts Box* Project: a qualitative exploration of the experiences, benefits, and challenges of participating in a remotely delivered art and craft project for British veterans with visual impairment

**DOI:** 10.1136/military-2022-002174

**Published:** 2022-09-07

**Authors:** Claire Castle, H Engward, T Kersey, L Kirk-Partridge

**Affiliations:** 1 Social & Welfare Research, BRAVO VICTOR, London, UK; 2 School of Music, University of Leeds, Leeds, UK; 3 Veterans and Families Institute for Military Social Research, Anglia Ruskin University, Chelmsford, UK; 4 Blind Veterans UK, London, UK

**Keywords:** complementary medicine, public health, qualitative research, social medicine

## Abstract

**Introduction:**

The clinical application of the arts among military personnel and veterans has been well documented, particularly in relation to service-related mental health difficulties. However, the impacts of engaging recreationally with art activities on general well-being remain underexplored and even more so among those living with visual impairment (VI). This pilot explored the artistic experiences of veterans with VI participating in a remotely delivered art and craft project during continued COVID-19 restrictions in Spring/Summer 2021.

**Methods:**

Six participants received a *mystery arts box* (*MAB*) containing a selection of materials, collated to encourage experimentation with unfamiliar techniques. Participants were asked to journal their process as they developed a final piece/pieces. They were invited to join group video calls to share work and ideas and seek guidance. Semistructured interviews were run with participants at the end of the project. Journal and interview data were thematically analysed.

**Results:**

Analysis identified 11 themes relating to initial and ongoing responses to the *MAB* and creative and journalling process. Several benefits were identified, including artistic learning, trying something new, and social, cognitive and emotional experiences. The value of the activity to participants’ lives within the context of the ongoing pandemic was also considered. Challenges were associated with the use of unfamiliar materials, impacts of sight loss and the limitations of remote delivery.

**Conclusion:**

This pilot brings to the fore the everyday artistic experience of veterans living with VI and considers the benefits, challenges and well-being implications of a remotely delivered arts activity. Findings illustrate the importance of ensuring accessibility of artistic activities to those for whom disability might limit participation and highlight the ongoing role that remotely delivered arts activities might play in meeting the social and recreational needs of individuals beyond the COVID-19 pandemic.

**What is already known on this topic**

- Literature documents the role that art therapy has played in the clinical treatment of military personnel and veterans, but the recreational artistic experiences of these individuals remain underexplored.
- The visual arts experiences of individuals with visual impairment, for whom sensory loss might be seen to limit engagement with some creative activities, have also seldom been considered.

**What this study add**

- The pilot is the first to consider the well-being implications of a remotely delivered art and craft activity for veterans with visual impairment.
- This pilot builds on existing knowledge generated within the field of art therapy, this time focusing on the role of non-clinical artistic experiences in the lives of veterans and also considering the impact of visual impairment on these experiences.

**How this study might affect research, practice and/or policy**

- Findings demonstrate the benefits and challenges associated with remote delivery models for arts activities to veterans with visual impairment, with implications for the design and delivery of future rehabilitative interventions and leisure activities.
- The pilot highlights the importance of representing the voices of veterans, including those with visual impairment, and the wider visually impaired population in visual arts research, and issues of accessibility that should be considered to ensure greater participation amongst these individuals in the future.

## Introduction

Literature documents the therapeutic application of activities such as drawing, photography, painting, quilting, crocheting, sculpting and mask-making to meet the clinical needs of military personnel and veterans.^1–3^ Outcomes relating to increased self-awareness, emotional expression, communication, self-regulation, problem-solving and shared experience have been demonstrated.^4^ However, there has been little consideration of the artistic experiences of these groups outside the clinical context. This is despite evidence of the well-being benefits of recreational arts participation in general populations.^5–7^ Activities such as textile craft, pottery and painting have been associated with a sense of achievement and personal growth, self-expression, the development of both physical and cognitive skills,^6 7^ stress reduction^8^ and social benefits.^9 10^

The artistic experiences of those living with visual impairment (VI) have also been overlooked, which may be partly explained by the problematic assumption that art making is undertaken to produce visual articles and, thus, is the domain of sighted individuals.^11^ Yet, art therapy research has found that clients with VI have used ‘visual’ arts methods to achieve a variety of outcomes.^12 13^ Art’s physical and tactile aspects may supplement, or supplant, the importance of visual perception for those with VI.^14^ However, few individuals with VI are completely blind, and activities such as textile/e-textile design,^15^ photography, sculpture^11^ and painting,^16^ remain accessible to many who retain some vision. These activities offer opportunities to take ownership of an artistic process and product, think and work independently, demonstrate persistence and problem-solving, spend time with others, and develop creative skills.^15^

## Current project

Originally planned for face-to-face delivery, the *Mystery Arts Box (MAB) Project* was redesigned for remote delivery during the COVID-19 pandemic. The pilot sought to generate knowledge surrounding the artistic experiences of military veterans outside the therapeutic context and to represent individuals with VI in visual arts research. This article addresses three questions:

1. What were the benefits of participation in the *MAB* Project for veterans with VI?
2. What were the challenges associated with participation in the *MAB Project* for veterans with VI?
3. What impact did VI have on participation in the *MAB* Project?

Designed as an exploratory pilot of an inherently subjective phenomenon, the study adopts a phenomenological view of arts engagement, acknowledging the subjectivity and qualitative diversity of human experience.^17^

## Methods

### Participants and recruitment

All six participants (mean age 73.6, ranging from 52 years to 89 years) were members of Blind Veterans UK BVUK, a charity which provides support to military veterans living with VI equivalent to ‘blindness’/severe sight impairment. Participants were recruited via an advert in the charity’s magazine and direct contact from the BVUK art and craft team.

### Procedure

Participants engaged with four activities: art making, journalling, group calls (*creative circles* (CCs)), and semistructured interviews. Participants received a mystery box of art and craft materials, and were asked to develop a piece/pieces and journal their creative process over 12 weeks. Four CCs were run via teams by the lead researcher with a member of the () art and craft team, offering opportunities for participants to seek assistance, share ideas and connect with other participants. At the end of the project, participants took part in a semistructured interview which was audio recorded and transcribed (using NVivo Transcription with manual quality checks).

### Data and analysis

Data were deidentified using pseudonyms, allowing journal and interview data and artwork, to be matched for each participant. Thematic analysis offered a practically and theoretically flexible tool with which to analyse journal and interview data.^18^ While themes were inductive, the researchers acknowledge the active role taken during the research process and the interpretation inherent in the identification of patterns within qualitative data.^17^ Initial coding was undertaken independently by three researchers to increase intercoder reliability; the lead researcher collated data and organised codes and themes using NVivo software. Artwork was not analysed, with the focus being on participants’ creative process and experiences, not the output itself. Final pieces acted as prompts during interviews.

### Materials

The content of the *MAB* was developed with the lead member of the art and craft rehabilitation team. The materials sought to encourage experimentation and tactile engagement. Materials included papers of different textures and thickness; paint pads, to encourage dipping/printing; and sponges, skeleton leaves and paper straws. Participants received an A3 sketchbook, resources for journalling (yellow journal pad or audio recording device), an ideas sheet and journalling guidance.

An interview schedule was used flexibly to guide discussion and ensure key topics were explored, including reasons for participating, enjoyable or challenging aspects of the project, and reflections on the CCs and materials, their creative process, and their final pieces.

## Results

Five out of six participants contributed journal data, with one participant forgetting to journal; this individual did not appear hugely interested in journalling and, due to personal circumstances, missed two of the CCs which would have acted as reminders to journal. Another participant (P5) agreed for personal correspondence with the lead researcher to be included in the data set. All participants took part in a semistructured interview. CC attendance varied due to participant availability. Table 1 provides an overview of themes and subthemes derived from journal and interview data.

**Table 1:** Overview of themes and sub-themes derived from journal and interview data

**New or unfamiliar materials**	**Expectations**	**Responses to the** ***MAB***	**Sight change**
Adding to the materials provided	What’s in the box	Positive reactions	‘Accessible’ materials
Familiar materials	Expectations of researcher or others	Excitement	Assistance from others
Putting other art activities on hold	Feeling encouraged	Surprise	Change in/limited colour perception
Benefits of unfamiliar materials	Expectations of self	Negative reactions	Not wanting to experiment
Adaptability	**Creative circles**	Disappointment	Past leisure activities
Experimentation with materials	Social	Lack of motivation	Positive impact on *MAB* experience
'Giving it a go', a new way of working	Sharing of art	Overwhelming	Tactile nature of materials
Skill development	Wanting feedback or help	Not enough guidance	Technology making art accessible
Negative reactions to new materials	A social outlet	No clear end point	VI as a limiting factor
Concern over mess	Learning from others	Not sure what to think or do	**Participants’ artwork**
Giving up	Shared experience	Importance of time	Feedback from others
Intimidated by new materials	Overwhelming	Not enough/too much time	Meaning attached to piece
Limited by physical space	Beliefs about own and others' abilities	Right amount of time	Positive self-assessment
When things go wrong	Feeling judged	Thinking time	Aesthetically pleasing
Walking away/waiting a while	Limitations of virtual delivery	Initial reactions to *MAB*	Colour, shape and composition
**Positive impacts on the individual**	Logistical and technology challenges	Planning and progress over project	Does not need to be aesthetically pleasing
A challenge	**Mood**	Comparison to others’ progress	‘At ease’ with medium
A new experience	Impact of art making on mood	Engagement changing over time	Negative self-assessment
Therapeutic	Expectations of researcher’s understanding of mood	**What is art?**	Quality, ‘bad’
Inspiring other creative activities (and vice versa)	Mental health	Art experience and knowledge	Sources of inspiration
Freedom	Depression	Colour	Artists and other artwork
Keeping busy (COVID-19 context)	**Journalling**	How is art talked about	Nature
**Negative impacts of participation**	Unsure what to journal	Imagination	What’s in the box
Regret	Methods of journalling	Individual	Instructions
		Self-expression through other art forms	The artistic process
			Planning sessions

MAB, mystery arts box; VI, visual impairment.

### Benefits of participation

#### Creative benefits

Participants enjoyed the opportunity to experiment using unfamiliar materials. This was an opportunity to approach art making in a new way.

P1 journal: It reminds me of a program I used to watch called Ready Steady Cook, where they gave them a big bag of food, they emptied the bag out on the table and then said, have five min to look at it and then make your mind up what you're going to cook.

The emphasis on experimentation led to a focus on ‘giving it a go’, rather than on how a final piece looked, along with the development of artistic skills and techniques.

P2 interview: When I’ve got something to do, to draw, or got an idea, I’ve now got more ways of doing it

With unforeseen challenges came unexpected positive outcomes, including the resourceful use of ‘waste’ products.

P4 interview: Newsprint should be able to take the ink, but this basically goes straight through it. Well guess that was good actually… the cardboard became a piece of work

#### Cognitive and emotional benefits

The project was viewed as a creative challenge, which pushed participants out of their comfort zone. This provided a welcome distraction during COVID-19: an opportunity to escape from negative thoughts or emotions and a positive focus at a time where usual activities were restricted.

P6 interview: It’s given me something to do I suppose which you do need at this time… I think that is necessary otherwise I don’t know what, you’d just go quietly crackers

The arrival of the *MAB* offered excitement and a break from routine, while art making itself was cathartic and a means to achieve affective change.

P1: It puts you in a good mood… you’d say right we’re going to get something done today and once you’d started… I found it was really relaxing

The therapeutic benefits of participation were perhaps most apparent for P5, who had struggled with his mental health and was open about using the project to address therapeutic goals.

P5 journal: I told you that all my work will all be connected with a story, well it is my struggle with [details of mental health difficulties]. The Mask is what I wore all happy and smiling on the outside but behind is what is really going on the real me. I know I’m probably taking liberties here but I’m using it to heal and sort of Art Therapy.

#### Social benefits

CCs provided opportunities to learn from each other and members of the BVUK art and craft team.

P2 interview: I find the chats quite interesting because you pick up tips from other people

Within the context of the COVID-19 pandemic, the opportunity for a shared experience may have been particularly important. Three out of six participants lived alone, and P6 highlighted the impact of isolation at this time, ‘I think I was getting depressed… I was alone 24 hours a day’.

P1 interview: Listening to other people as well… there’s only a couple of people that phone me in normal times and it’s usually doom and gloom, you know… I looked forward to them [CCs].

### Challenges associated with participation

#### Concerns over mess and managing materials at home

While new materials provided creative possibilities and opportunities to hone new skills, they also resulted in challenges. There were concerns over mess and managing materials, particularly where space was limited or there was another person living at home. Exploring new techniques might generate greater mess than familiar activities.

P1 interview: I’ve got a wee bench of sorts and mess doesn’t matter as long as I clean myself down afterwards… its different outside, if it was inside in your kitchen or something, I do a lot of painting in my kitchen, but the painting’s different.

Concerns over mess resulted in ‘giving up’ on materials, or complete avoidance from the outset.

#### Initial responses to the *MAB* and feeling overwhelmed

While there were feelings of excitement associated with receiving the *MAB*, some participants felt initially overwhelmed by the task at-hand.

P2 interview: I was afraid to use them [some of the materials] quite honestly …it does intimidate you when you’re faced with something totally outside your comfort.

In contrast, P5 reported positivity on the arrival of the *MAB*, but soon felt unsure of how to proceed. A lack of creative guidance may inhibit some.

P5 journal: After first feelings of excitement I now feel nothing. I don’t know what I’m going to do

#### Negative social impacts of CCs project design

P5 felt that the CCs had not provided the support he sought, leading to negative self-comparison due to what he viewed as his own artistic inadequacies:

P5 interview: He [another participant] did his classes and doing all this, and I sat there thinking, I can't live up to that, I can’t

This, alongside his comparative younger age and ongoing mental health difficulties, led to feelings of exclusion, ‘I was probably the youngest one… I just felt from the onset I felt like an outsider’. P2 came to the project with a similarly low level of artistic experience, but his response to the achievements of others was driven by admiration, rather inadequacy: ‘They were blooming clever, a lot cleverer than I was’.

P4 highlighted that without equal participation across the group, learning may be limited, ‘I didn’t learn much from the others… [they] had the box for a month and hadn’t even looked at it’. Again, the flexibility afforded to participants may not always benefit their engagement.

#### Technological challenges and limitations of remotely delivered arts activities

To ensure accessibility of *CC*s, participants were able to join by video call or by phone. However, this limited the engagement of those who joined only by phone.

P6 interview: I did wonder what these things looked like I think one man had made a model or something or rather and you said oh yes, those little stick man and he said, no they're trees.

Across the group, the virtual delivery of the sessions was perceived as less effective and less enjoyable than activities run in person; the lack of instant feedback was a limiting factor.

P1 journal: I wouldn't mind having somebody in the house to knock ideas about with, but unfortunately that’s not the way it is.

Furthermore, after over a year of social restrictions and altered service provision, P4 felt saturated in remotely delivered projects.

P4 interview: There’s been so much remote sort of stuff in the last year and a half, it’s alright doing this, but obviously we like to have instant feedback.

### Impacts of VI on participation

#### Art making with a VI

Participants reflected on the negative impacts of sight loss on art making, including a loss of ability to see detail, and difficulty drawing, cutting out and differentiating colours. P1 noted that VI may make the risk of ‘mess’ much higher, particularly when working alone.

P1 journal: [Ink] it’s messy for somebody with a sight problem who hasn’t got anybody here to see and point out where I've dripped a bit.

Participants had found solutions to some of their difficulties, including the use of alternative techniques and magnification technology to enlarge details. P2 sought assistance from his partner, although this support was not always available.

P2 interview: If I wanted to use some particular colours then I would I call my partner to pull them out for me… Of course they’re not available all the time, I mean she’s got her own life to lead… I didn’t like to bother her too much

#### Positive impacts of sight loss on the *MAB Project* experience

Despite the challenges, participants used various new materials and techniques. P5 even suggested that the initial experience of exploring the materials may have been heightened by having a VI.

P5 interview: What I think is the most magical thing about it, is if I was sighted, and I know this is a point of it, is it would have been completely different… it makes it a bit more exciting, you can’t see it for what it is.

Some items were viewed especially useful to art makers with a VI. P2 highlighted the benefits of tactile items such as bamboo straws and corrugated paper, the latter of which had provided a solution to difficulties that multiple participants reported with cutting out.

P2 interview: It was tactile, that was the main thing with it… I could actually feel the shape I wanted to cut… I knew roughly how big I wanted it, and after a bit of trial and error, I could count the ridges in the corrugated paper.

Activities which encourage tactile engagement with art materials may offer opportunities for individuals with VI to find solutions to challenges experienced during art making.

## Discussion

Participation was associated with emotional, cognitive and social benefits. Findings reflect those of existing literature, which highlights the well-being outcomes associated with arts participation among both the general population and specific groups,^6^ including older adults and those living with chronic illness and/or disability.^19 20^ The psychological benefits of participation may have been particularly important during the COVID-19 pandemic, with this period being associated with increased psychological distress for many.^21^ Other research carried out at this time found that activities such as drawing, textile art and photography provided individuals with a positive distraction from current events and a sense of purpose.^22^ Similar to the current study, Armstrong and Ross provided art boxes to parents and children during lockdown to promote connection through playful, creative shared experiences.^23^ While the current pilot did not allow for participants to make art together, the activity offered a shared artistic experience, and opportunities to learn from others and feel socially connected. Vogelpoel and Jarrold suggest that art making opportunities may be a driving factor in renegotiating experiences of inclusion and social participation for individuals with VI.^24^ This may be increasingly important during a return to ‘normal’ recreational life following the pandemic.

Regardless of sight loss, participants experimented, to some degree, with unfamiliar materials. There was even a suggestion from one participant that VI might increase enjoyment of using unknown, tactile materials. Research suggests that tactile materials may be more useful to individuals with VI than techniques such as painting or drawing,^14^ although techniques including larger-scale working, avoidance of fine visual/motor work, and use of additional sensory elements such as scent, might increase the accessibility of art and craft activities for individuals with VI.^15 25^ These factors may be valuable considerations for the design of future projects.

Participants also experienced challenges, both related and unrelated to their VI. Experimentation and the use of unfamiliar materials led to mess, which for individuals with VI, especially those living alone, may be a particular concern. While a lack of goal-setting, and an emphasis on creative freedom offered autonomy, some felt unsure of how to proceed. There was also reflection on the practical and creative challenges of remotely delivered art activities, including the limitations of home working (eg, a lack of assistance or instant feedback). Art making in the vicinity of others was considered not only a social activity but also beneficial to skill development.

### Limitations and future research

The current project was a pilot with six self-selected participants from BVUK. This limits the extrapolation of findings to either the wider veteran community, or others with VI. The *MAB Project* was a product of the COVID-19 context, and it remains to be seen if the same benefits and challenges would exist beyond this. Future research is needed to consider the efficacy of both remote and face-to-face arts activities to meet the goals of participants with a wider sample of veterans, including those living with VI, beyond the pandemic. Further consideration of the social implications of art making alone, or with others, for veterans with and without VI may be particularly useful. Further qualitative and quantitative research is needed to understand the impacts of different art activities on the health and well-being of veterans, and the influence of factors such as age, veteran status and nature of VI on recreational art participation.

## Conclusion

This article provides insight into the experiences, benefits and challenges associated with participation in a novel, remotely delivered art and craft activity for six British veterans with VI. Findings highlight that art and craft activities may be a valuable tool to address everyday psychological needs of veterans with VI, and that remote delivery may be a useful alternative to face-to-face delivery. However, challenges may arise, particularly if working with individuals whose access to, or knowledge of, technology is limited; face-to-face delivery will likely remain preferable for many. The current study confirms Szubielska’s belief that ‘By engaging in visual arts, people with sight impairments show that the artistic creative process can be altered. They prove art is not reserved for sighted people only’ (Szubielska, p1535).^11^ Findings demonstrate the contribution of recreational arts experiences, including hobby groups and self-directed art making, to health and well-being outcomes.

10.1136/military-2022-002174.supp1 Supplementary data

## Data Availability

Data are available upon reasonable request.
